# Vitamin D level and its association with adiposity among multi-ethnic adults in Kuala Lumpur, Malaysia: a cross sectional study

**DOI:** 10.1186/s12889-016-2924-1

**Published:** 2016-03-07

**Authors:** I. S. Shafinaz, F. M. Moy

**Affiliations:** Julius Centre University of Malaya, Department of Social & Preventive Medicine, Faculty of Medicine, University of Malaya, 50603 Kuala Lumpur, Malaysia

**Keywords:** 25(OH)D, BMI, Body fat percentage, Waist circumference, Physical activity, Sun avoidance

## Abstract

**Background:**

Vitamin D deficiency is highly prevalent in both temperate as well as tropical countries. Obesity is one of the factors contributing to vitamin D deficiency. As our country has a high prevalence of overweight and obesity, we aimed to study serum 25-hydroxyvitamin D (25(OH)D) level and its association with adiposity using various adiposity indicators; and to study other risk factors that affect serum 25(OH)D level among multi-ethnic adults in Kuala Lumpur, Malaysia.

**Methods:**

This was a cross sectional study conducted with a multistage sampling. All permanent teachers working in government secondary schools in Kuala Lumpur were invited for the study. The data collection included serum 25(OH)D, Parathyroid Hormone (PTH), body fat percentage, waist circumference, body mass index (BMI) and blood pressure. Demographic characteristics, sun avoidance, sun exposure and physical activity were enquired from the participants using a self-administered questionnaire. The data was analyzed using a complex sample analysis.

**Results:**

A total of 858 participants were recruited. Majority of them were Malays, females and had tertiary education. The overall prevalence of vitamin D deficiency (<20 ng/ml) was 67.4 %. Indian participants (80.9 %) had the highest proportion of vitamin D deficiency, followed by Malays (75.6 %), others (44.9 %) and Chinese (25.1 %). There was a significant negative association between serum 25(OH)D level with BMI (*β = −*0.23) and body fat percentage (*β =* −0.14). In the multivariate linear regression analysis, Malays, Indians and females (*p* < 0.001); higher BMI and larger waist circumference (*p* < 0.05) were significantly associated with lower serum 25(OH)D level. The full model explained 32.8 % of the variation between participants in the serum 25(OH)D level. The two most influential factors affecting serum 25(OH)D level were ethnicity and gender.

**Conclusions:**

The prevalence of vitamin D deficiency among our participants was high. Adiposity was associated with serum 25(OH)D level. Skin pigmentation and gender based behaviours were more dominant in contributing to serum 25(OH)D level. Health education should be targeted in weight management, gender based behaviours on sun exposure, as skin pigmentation is non-modifiable.

## Background

Vitamin D plays an essential role in health. Its deficiency increases the risk of osteoporosis as well as contributes to cardiovascular diseases, diabetes and cancers [1–3]. Skin synthesis of vitamin D from sunlight exposure is the major source of vitamin D [4]. Evidences show that temperate countries, as well as tropical countries like Malaysia, Thailand, Saudi Arabia and Iran [5–8] experience the problem of vitamin D deficiency.

Vitamin D status can be measured using 1,25-dihydroxyvitamin D (1,25(OH)_2_ D) and 25-hydroxyvitamin D (25(OH)D). However, the circulating 25(OH)D is considered a better marker to determine vitamin D status as it has a long circulating half-life of 14 to 20 days [9]. In contrast to 25(OH)D, 1,25(OH)_2_D is less used to determine vitamin D status due to its short half-life (approximately 15 hours). Its serum concentration is also closely regulated by parathyroid hormone (PTH), phosphate and calcium [10]. In addition, 1,25(OH)_2_D level does not typically decrease until there is a severe vitamin D deficiency [11, 12].

Malaysia is an upper middle income country and its prevalence of obesity is high. According to the National Health Morbidity Survey in 2011, the prevalence of overweight and obesity among adults aged 18 years and above was 29.4 % and 15.4 % respectively [13]. One of the factors contributing to vitamin D deficiency is obesity. Obesity-associated vitamin D deficiency is most likely due to the decreased bioavailability of vitamin D with the deposition of vitamin D in the adipose tissue [14]. It is also hypothesized that a reduced circulating vitamin D in the form of calcidiol stimulates an accumulation of fat mass, giving rise to obesity and the induction of metabolic syndrome. It may be possible to reverse the condition of obesity by improving vitamin D status [15]. Based on this evidence, it is expected that obese individuals may need higher than the usual doses of vitamin D. However, it remains unclear of which indicator of adiposity, i.e., body mass index, waist circumference or percentage of body fat that should be taken into consideration while assessing vitamin D status in the general population.

Body Mass Index (BMI) is commonly used to determine one’s healthy weight with reference to the individual’s height. Many studies used BMI to measure obesity [5, 16] as it is simple, effective and quick to be applied on adults and children. Waist circumference is used to estimate visceral fat or central obesity. However, both of these indicators are indirect measures of body fat. Body fat percentage, which is a direct measure of total body fat, can be measured by the Dual Energy X-ray Absorptiometry (DEXA) or the Bioelectrical Impedance Analyzer (BIA).

There is a scarcity of published reports on the association of adiposity and vitamin D status among the adult population in Malaysia. To date, the only available Malaysian studies on vitamin D amongst adults are the vitamin D status among postmenopausal Malaysian women [16]; the status of vitamin D among women with child bearing-age [17]; the vitamin D status on obesity, metabolic syndrome and cardio-metabolic risks among females in Kuala Lumpur [18]; the effects of nutrition education and sun exposure on vitamin D status among postmenopausal Malay women which was extended from Rahman’s study [19]; the effects of sun exposure on 25(OH)D concentration among urban and rural women in Kuala Lumpur and Negeri Sembilan [20] and the latest study reported the vitamin D status among Malaysian men and its associated factors [21]. Among these, two studies used the BIA to relate body fat percentage with vitamin D status [20, 21]. However, that association was not the researchers’ main focus [20] and the study by Chin et al. was only limited to men [21], while the rest of the studies used BMI, an indirect measurement of adiposity.

Therefore, we aimed to study the association of serum 25(OH)D level and adiposity using various adiposity indicators and to study other risk factors that affect serum 25(OH)D level among multi-ethnic adults in Kuala Lumpur.

## Methods

### Study design

This was a cross sectional study, conducted from February to May 2013 and it was part of the CLUSTer cohort study [22].

### Study population

All permanent teachers who worked in the selected government secondary schools in Kuala Lumpur were invited to participate. Teachers were selected because they are easily accessible during working hours and they are one of the largest group of workers in the country.

### Sampling method

A two stage sampling method was carried out. First, 50 % of all government secondary schools (*n* = 87) from all districts (*Pudu, Bangsar, Sentul, Keramat*) in Kuala Lumpur were randomly selected. Then, all teachers from the selected schools, except those who were pregnant, were invited to participate in the study. Pregnant teachers were excluded as their levels of adiposity and requirement for vitamin D may be different from those who were not pregnant.

### Ethics clearance, approval and informed consent

Ethics clearance was obtained from the University Malaya Medical Centre (UMMC) Ethics Committee (Reference Number: 950.1) that governs all studies on human within the Medical Faculty. Approval from the Ministry of Education, Malaysia; the Education Board of Wilayah Persekutuan Kuala Lumpur and the principals from each selected schools were obtained before data collection. Informed consent was obtained from all participants.

### Data collection

The data collected included 1) serum 25(OH)D and PTH, 2) fat percentage using the Bioelectrical Impedance Analyzer (BIA), 3) anthropometric measurements (BMI and waist circumference) and 4) blood pressure. A fasting venous blood sample was collected for serum 25(OH)D and PTH analysis. The blood samples were spun at 3000 revolutions per minute (RPM) for 15 minutes. Then, the serum was separated and stored in a −80 °C freezer until the analyses of vitamin D and PTH were carried out. Electro-chemiluminescence immunoassay (ECLIA) on the Cobas E-411 analyzer was used to analyse serum 25(OH)D. The inter-assay coefficient of variation (CV) was 3.6 % at 22.8 ng/ml and 3.0 % at 68.2 ng/ml, while the intra-assay CV was 3.5 % at 22.8 ng/ml and 2.9 % at 68.2 ng/ml. The measuring range for this kit was 4 to 100 ng/ml. Serum PTH was analyzed using the ECLIA PTH on the Cobas E-411 analyzer. The inter-assay coefficient of variation (CV) was 6.2 % at 2.14 pmol/L and 4.1 % at 6.15 pmol/L, while the intra-assay CV was 4.1 % at 2.14 pmol/L and 2.2 % at 6.15 pmol/L. The measuring range of this kit was 0.127 to 530 pmol/L.

Serum 25(OH)D less than 20 ng/ml or 50 nmol/l was considered as vitamin D deficient, according to the US Endocrine Society Clinical Practice Guidelines [23]. PTH was measured along with 25(OH)D as both are responsible for maintaining extracellular calcium homeostasis [24] and to rule out vitamin D deficiency due to hyperthyroidism.

Height was measured using a stadiometer (Seca, Germany). Body fat percentage and weight were measured using the foot-to-foot Bioelectrical Impedance Analyzer (Tanita TBF-300A) and a weighing scale (Seca, Germany) respectively, with socks and shoes removed. Body mass index (BMI) was calculated as weight in kilograms divided by the square of height in meters. Following the recommendations of World Health Organization for Asians [25], a BMI of less than 17.5 kg/m^2^ was categorised as underweight and BMI in the range of 17.5 to 22.9 kg/m^2^ was healthy/normal. BMI of 23.0 to 27.9 kg/m^2^ was considered as overweight, whereas BMI above or equal 28.0 kg/m^2^ was categorised as obese. The waist was measured at the midpoint between the lower costal margin (lower rib) and the iliac crest (top of the pelvic bone) [25, 26]. Waist circumference of more and equal to 90 cm and 80 cm for men and women respectively was considered at risk or abnormal. Blood pressure was measured using the clinical validated digital automatic blood pressure machine (OMRON HEM Model- 907) while the participants were in a seated position.

Socio-demographic characteristics such as age, gender, ethnicity, religion and marital status were enquired. A validated questionnaire on sun exposure and avoidance practices was administered. Participants were asked about their outdoor activities and clothing styles like wearing long sleeves, long skirts, hat/cap, veils, and use of umbrellas and sun block lotions. Sun avoidance score was derived through the sum of all types of avoidance as mentioned above (max = 8, min = 0), while sun exposure score was derived by multiplying the duration in minutes of sun exposure per day with the number of days per week [18]. Physical activity was assessed using the validated Malay International Physical Activity Questionnaire (IPAQ-M) short form [27]. All continuous scores were expressed in MET-minutes/week. For the calculation of IPAQ-M data, the following MET-values were used: walking = 3.3 METs, moderate physical activity = 4.0 METs and vigorous physical activity = 8.0 METs. The MET-minutes per week (MET-min week^-1^) was calculated as minutes of activity/day x days per week x MET level. Finally, total physical activity MET-minutes/week was calculated as the sum of walking + moderate + vigorous MET-minutes/week scores.

### Data analysis

Statistical analysis was performed using the Statistical Package for Social Sciences for Windows, version 22.0 (SPSS Inc., Chicago, IL, USA). Weighted means ± standard deviation (SD) and proportions were calculated for descriptive data. Complex Sample Analysis was used. Final weightage was calculated by multiplying schools’ weightage and teachers’ weightage. Weightage was used in the Complex Sample Analysis to correct the unequal probabilities of non-respondence.

Independent t-test, analysis of variance (ANOVA), simple linear regression and multiple linear regression analysis were conducted. Independent t-test and ANOVA (Table 2) were performed to analyse each categorical risk factor that influenced the mean serum 25(OH)D level. Simple linear regression analysis (Table 3) was performed to determine the association of the factors with serum 25(OH)D level, whereas multiple linear regression analysis (Table 4) was performed to determine the factors associated with serum 25(OH)D level after being adjusted for the other confounders. *R* ^2^ values were reported to compare the variation explained by each multivariate model. The significant level was preset at 0.05.

## Results

A total of 858 teachers from 30 schools in all four districts (*Pudu, Bangsar, Keramat* and *Sentul*) from Kuala Lumpur participated in this study. The response rate of teachers was 41 %. Table 1 shows the baseline characteristics of the participants. The majority of them were females (90.9 %), married (84 %) and within the age range of 30–49 years (66.5 %). Most of them were Malays (74.2 %), followed by Chinese (16.4 %), Indians (8.3 %) and others (0.4 %).

**Table 1:** Socio-demographic characteristics (*N* = 858) of participants

Characteristic	*N* (Weighted %)
Districts	
Bangsar	236 (28.2)
Keramat	255 (21.1)
Pudu	253 (24.8)
Sentul	114 (25.9)
Gender	
Male	77 (9.1)
Female	781 (90.9)
Age (years)	
< 30	137 (15.9)
30–39	279 (31.6)
40–49	293 (34.9)
≥ 50	149 (17.6)
Ethnicity	
Malay	660 (76.9)
Chinese	125 (16.4)
Indian	63 (8.3)
Others	10 (0.4)
Religion	
Islam	666 (75.3)
Buddhist	99 (12.8)
Hindu	53 (6.8)
Christian	34 (4.5)
Others	6 (0.5)
Marital status^b^	
Single	103 (14.6)
Married	611 (84.0)
Divorced/Widowed	14 (1.4)
Body mass index (kg/m^2^)^a^	
Underweight (<17.5)	32 (4.1)
Normal (17.5–22.9)	316 (36.4)
Overweight (23.0–27.9)	207 (24.2)
Obese (≥28.0)	303 (35.3)

^a^ Classification according to World Health Organization for Asians

^b^ *N* = 728

The weighted means for serum 25(OH)D and PTH were 17.97 ± 7.32 ng/ml and 6.13 ± 3.74 pmol/L respectively. A total of 578 participants (67.4 %) had vitamin D deficiency (<20 ng/ml). Indian participants (80.9 %) had the highest proportion of vitamin D deficiency, followed by Malays (75.6 %), others (44.9 %) and Chinese (25.1 %). Among those with vitamin D deficiency, more than half of them were obese (39 %) and overweight (24 %) (data not shown). Females had a significantly lower weighted mean serum 25(OH)D level compared to males (Table 2). Weighted means of serum 25(OH)D level were lowest among the participants of Indian ethnicity, aged <30 years, obese (≥28.0 kg/m^2^) and with higher body fat percentages (Tertile 3).

**Table 2:** Weighted mean serum 25(OH)D level of socio-demographic characteristics and adiposity indicators

Characteristics	Serum 25(OH)D	*p*-value
	Weighted mean, $\overline{x}\left(95\%\ \mathrm{C}\mathrm{I}\right)$	
Age (years)		
< 30	16.76 (15.56 to 17.97)	0.001
30–39	17.24 (16.36 to 18.11)	
40–49	17.81 (16.86 to 18.78)	
≥ 50	20.69 (19.11 to 22.27)	
Gender		
Male	25.27 (23.41 to 27.14)	<0.001
Female	17.24 (16.68 to 17.80)	
Ethnicity		
Malay	16.57 (16.06 to 17.09)	<0.001
Chinese	25.44 (23.84 to 27.05)	
Indian	15.47 (13.98 to 16.96)	
Others	19.55 (14.18 to 24.92)	
BMI (kg/m^2^)		
Underweight (<17.5)	17.29 (14.15 to 20.43)	<0.001
Normal (17.5–22.9)	19.20 (18.23 to 20.17)	
Overweight (23.0–27.9)	18.30 (17.02 to 19.57)	
Obese (≥28.0)	16.56 (15.82 to 17.30)	
Tertile BF %		
Tertile 1 (≤24.77)	18.92 (17.58 to 20.27)	0.037
Tertile 2 (24.78–31.62)	16.93 (15.83 to 18.02)	
Tertile 3 (≥31.63)	16.90 (15.92 to 17.88)	
Waist circumferences (cm)		
Male		
< 90 cm	26.06 (23.54 to 28.57)	0.227
≥ 90 cm	23.83 (21.20 to 26.46)	
Female		
< 80 cm	17.80 (17.01 to 18.59)	0.023
≥ 80 cm	16.51 (15.74 to 17.29)	

The weighted means of fat percentage and BMI were 28.1 ± 8.0 % and 25.66 ± 5.06 kg/m^2^ respectively. A total of 303 participants (35.3 %) were obese (≥28.0 kg/m^2^), while 207 (24.2 %) were overweight. Among those who were obese, 222 (74.4 %) had vitamin D deficiency. In the univariate analysis, BMI (*β* = −0.23, *p* < 0.001) and fat percentage (*β* = −0.14, *p* < 0.005) were negatively associated with serum 25(OH)D level, but not waist circumference (*p* > 0.05). The level of PTH and the sun avoidance score were also negatively associated with serum 25(OH)D level (*p* < 0.001), while age (*p* < 0.001) and systolic blood pressure (*p* < 0.05) showed positive association with serum 25(OH)D level (Table 3).

**Table 3:** Risk factors of serum 25(OH)D level among the participants

	Serum 25(OH)D	*p*-value
	Unstandardized *B* ± SE	
Age	0.13 ± 0.03	<0.001
Gender	−3.91 ± 0.38	<0.001
Ethnicity		
Chinese	Reference	
Malay	−8.87 ± 0.86	<0.001
Indian	−9.98 ± 1.12	<0.001
Others	−5.90 ± 2.85	0.039
Body mass index (BMI) (kg/m^2^)	−0.23 ± 0.05	<0.001
Fat percentage (%)	−0.14 ± 0.04	0.002
Waist circumference (cm)	−0.32 ± 0.02	0.152
PTH (pmol/L)	−0.44 ± 0.09	<0.001
Physical activity (METs/min)	1.17 ± 0.33	0.929
Sun avoidance score	−1.03 ± 0.27	<0.001
Sun exposure score	0.03 ± 0.53	0.955
Systolic blood pressure	0.03 ± 0.02	0.029
Diastolic blood pressure	0.02 ± 0.02	0.334

Gender: Males = 1, Females = 2

Table 4 shows the multiple linear regression analysis of all the risk factors affecting serum 25(OH)D level. Model 1 (Reference) comprised of all the significant factors in the univariate analysis (except PTH) without being adjusted for adiposity indicators, while Models 2, 3 and 4 comprised of Model 1 + BMI, Model 1 + waist circumference and Model 1 + body fat percentage respectively. PTH was excluded in the analysis as it was an outcome of serum 25(OH)D level. By comparing all models that were adjusted for various adiposity indicators, there was not much difference in *R* ^2^ between each of the adiposity indicators (BMI, waist circumference and body fat percentage). There was comparable *R* ^*2*^ value in the models with BMI or waist circumference (*R* ^*2*^ = 33.0 %) and it was slightly lower in the model with fat percentage (*R* ^*2*^ = 32.3 %).

**Table 4:** Different adiposity indicators affecting serum 25(OH)D level after being adjusted for age, gender, ethnicity, sun avoidance score and systolic blood pressure ^a^

	Unstandardized *B*			
	Model 1	Model 2	Model 3	Model 4
Age	0.022	0.033	0.030	0.005
Gender	−7.485**	−7.461**	−7.918**	−7.426**
Ethnicity				
Chinese	Reference	Reference	Reference	Reference
Malays	−9.030**	−8.512**	−8.763**	−9.046**
Indians	−9.550**	−9.070**	−9.138**	−9.241**
Others	−5.473	−4.965	−5.289	−2.845
Sun avoidance score	−0.047	−0.081	−0.055	0.413
Systolic blood pressure	0.001	0.013	0.011	0.011
BMI		−0.140*		
Waist circumference			−0.052*	
Fat percentage (%)				−0.016
*R* ^2^	32.0 %	32.8 %	32.6 %	32.3 %

^a^ PTH was excluded in the analysis as it is an outcome of serum 25(OH)D level

** *p* < 0.001, *p < 0.05

Gender: Males = 1, Females = 2

Model 1 = Controlled for age, gender, ethnicity, sun avoidance score, systolic blood pressure

Model 2 = Model 1 + BMI

Model 3 = Model 1 + Waist Circumference

Model 4 = Model 1 + Fat Percentage (%)

Among all the adiposity indicators, only BMI and waist circumference (*p* < 0.05) were significantly associated with serum 25(OH)D level after adjusted for all the confounders. The other factors significantly associated with serum 25(OH)D level were Malays, Indians and females (*p* < 0.001).

Our results demonstrated that adiposity was not the main risk factor that affected serum 25(OH)D level. The difference in *R* ^2^ between the reference model and the model adjusted for adiposity indicators (BMI and waist circumference) was only approximately 1.0 %. Table 5 shows that the more influential factors affecting serum 25(OH)D level were ethnicity and gender, as the difference in the *R* ^2^ values were 16.9 % and 7.1 % respectively.

**Table 5:** Contributions of gender and ethnicity to *R* ^2^ for serum 25(OH)D level

	Unstandardized *B*		
	Model 1 (Reference Model)	Model 2	Model 3
Age	0.033	0.022	0.138**
Sun avoidance score	−0.081	−0.638*	−0.377
Systolic blood pressure	0.013	0.025	0.021
BMI	−0.140*	−0.145*	−0.327**
Ethnicity			
Chinese	Reference	Reference	
Malays	−8.512**	−8.317**	
Indians	−9.070**	−9.558**	
Others	−4.965	−5.913*	
Gender	−7.461**		−7.269**
*R* ^2^	32.8 %	25.7 %	15.9 %
Difference in *R* ^2^		7.1 %	16.9 %

** *p* < 0.001, *p < 0.05

Gender: Males = 1, Females = 2

Model 1 = Predictors: age, sun avoidance score, systolic blood pressure, BMI, ethnicity, gender

Model 2 = Model 1 - Gender

Model 3 = Model 1 - Ethnicity

Difference in *R* ^2^ = *R* ^2^ in Model 1 (Reference Model) - *R* ^2^ in Model 2/Model 3

## Discussion

Based on the report from the Ministry of Science Technology and Innovation (MOSTI) in 2011, Malaysians received at least six hours of sunshine daily. However, many studies showed a high prevalence of vitamin D deficiency among the Malaysian population. Similarly, we found a high proportion of our participants had vitamin D deficiency (<20 ng/ml). Females had lower levels of vitamin D compared to males, similar as the previous findings [5, 28]. This could largely be explained by their clothing styles such as wearing veils, long sleeves, long skirts, using umbrellas and sunblock lotion. Our findings showed that there was a negative association between serum 25(OH)D level and sun avoidance score (*p* < 0.001) in the univariate analysis. However, only gender remained significant in the multivariate analysis. Cultural perception among Asians, especially the females’ preference for fairer skin [29, 30] may influence them to avoid the sun, have clothing styles that covered most parts of their bodies or use sun block lotion when going outdoor.

Similar with the study by Nurbazlin’s et al. [20], higher proportions of Indian and Malay women from our study had vitamin D deficiency compared to Chinese. This could be due to the darker skin color of Indians (Fitzpatrick skin type VI) and Malays (types V and VI) compared to Chinese (types III and IV) [31]. Higher melanin content in dark skin inhibits vitamin D synthesis [16]. Tsiaras et al. reported that those with darker skin pigmentation or higher melanin content required longer sun exposure compared to those with lighter skin to produce the same amount of vitamin D level needed in the body [32]. Ethnicity was significant in both univariate and multivariate analyses.

Lower vitamin D level was more prevalent with advancing age [33, 34]. Aging decreases the skin’s capacity to produce vitamin D [35]. Among the elderly, there is a decreased hydroxylation of vitamin D and response towards the intestinal mucosa to circulate vitamin D [36]. However, there are also studies reported that serum 25(OH)D level did not decrease with age [16, 30]. A study from Iran reported that 25(OH)D level remained constant from the age of 20 to 60 years among men [37]. Our results showed that older participants had higher serum 25(OH)D level compared with the younger participants. This could be due to younger individuals perceiving fair skin as a symbol of high social class and attractiveness [30]. As a result, they avoided being exposed to sunlight. In addition, our participants were in the age range of 30 to 60 years old, where they were relatively healthy compared to those who were older. Further studies should be carried out to explore the serum 25(OH)D level among adults over a wider spectrum of age.

Obesity is a well established risk factor for cardiovascular diseases [26, 38, 39], which increase morbidity and mortality in both developed and developing countries [25]. Similar with the previous findings [5, 40–43], obesity was significantly associated with serum 25(OH)D level. This may be explained by the characteristics of vitamin D itself as a fat soluble vitamin. Higher body fat reduces the availability of circulating 25(OH)D [16]. Obese individuals have higher fat content, which might block 25(OH)D from being sequestered into the body and eventually lowers the circulating serum 25(OH)D. We found BMI (*p* < 0.001) and body fat percentage (*p* < 0.05) to be negatively associated with serum 25(OH)D level in the univariate analysis. However, in the multivariate analysis, body fat percentage became insignificant, but higher BMI and larger waist circumference were significantly associated with lower serum 25(OH)D level (*p* < 0.05).

There were controversial results in the association of body fat percentage with serum 25(OH)D level. Arunabh et al. found that serum 25(OH)D level were more strongly associated with body fat percentage compared to BMI, indicating that it was adiposity, not simply body mass that influenced the serum 25(OH)D level [44]. However, we found contradicting results, as similarly reported by Nurbazlin et al. and Chin et al. [20, 21]. This could be due to the foot-to-foot BIA machine that may not be as accurate as the direct segmental multi-frequency (DSM)-BIA. In addition, our participants might not adhere to certain conditions to be followed such as fasting, avoid eating or drinking, or avoid exercising before measurements.

The present study found an inverse association between waist circumference and 25(OH)D level after adjusted for all confounders, as similarly reported by Lu’s et al. [45]. Central obesity has been linked with the increase in cardiovascular risks, development in hyperinsulinemia, insulin resistance, heart disease and high blood pressure [46] that might affect serum 25(OH)D level. Therefore, it is important to measure both the overall and central obesity while accessing vitamin D status and its cardiovascular risks in the future.

Parathyroid Hormone (PTH) is a hormone maintaining normal serum concentrations of calcium and phosphate. It is regulated through the levels of serum vitamin D and calcium [47]. Vitamin D deficiency is generally associated with an increase in PTH [48–50], as similarly found in our results. An elevated PTH concentration is known to be associated with cardiometabolic diseases [51, 52]. Therefore, there is a need to monitor both levels of serum 25(OH)D and PTH. On the other hand, some studies showed no significant association between 25(OH)D level and PTH [7, 17, 20, 30]. Serum calcium may be another factor that influences the level of PTH. Further investigation should be carried out on this aspect.

We did not find physical activity to be associated with serum 25(OH)D level. However, there were studies that reported significant association between physical activity with vitamin D level [16, 53]. Individuals who exercised may be exposed to more sunlight which contributed to the synthesis of vitamin D [54]. However, it remained unclear whether doing physical activity or exercise itself contributed to the elevated serum 25(OH)D level [53], or due to sun exposure from the exercise.

Among the significant factors associated with serum 25(OH)D level such as ethnicity, gender and BMI, we found that the contribution of BMI to the *R* ^*2*^ was only 0.8 %. The two most influential factors affecting serum 25(OH)D level were ethnicity and gender. Ethnicity as the proxy of skin pigmentation and gender based behaviours were more dominant in contributing to serum 25(OH)D level. Health education should be targeted in these gender based behaviours, as skin pigmentation is non-modifiable.

There are some limitations that warrant some discussion. First, there are various methods of determining serum 25(OH)D which may influence the readings of 25(OH)D level. Some of the immunoassays underestimate 25-hydroxyvitamin D metabolites due to the differences in the affinity between the antibodies or D-binding protein employed. Secondly, no gathered information on the medications and vitamin D supplements used among participants was another limitation. Recall bias from participants may also influence our results.

Notwithstanding the above limitations, the present study had several strengths. We tested the associations of multiple adiposity indicators with serum 25(OH)D level. Our sample comprised of multi-ethnic adults, thus making it possible to infer to all adults in Kuala Lumpur. We also established gender based behaviours as one of the important factors influencing serum 25(OH)D level. Health education and promotion programs should be targeted among the females who are at high risk of vitamin D deficiency. Efforts should be targeted in the methods to acquire more vitamin D. Further studies are required to ascertain if higher vitamin D is required for this population.

## Conclusions

Our participants had a high prevalence of vitamin D deficiency. In the multivariate analysis, Malays, Indians, females, higher Body Mass Index (BMI) and larger waist circumference were significantly associated with lower serum 25(OH)D level. Skin pigmentation and gender based behaviours were more dominant in contributing to serum 25(OH)D level. Health programs should be carried out to educate the community on how to prevent vitamin D deficiency in the future.
